# NICE medical technologies guidance: aims for clinical practice

**DOI:** 10.1186/2047-0525-2-15

**Published:** 2013-07-03

**Authors:** Bruce Campbell

**Affiliations:** 1 Medical Technologies Advisory Committee, National Institute for Health and Clinical Excellence, Manchester M1 4BD, UK

## Abstract

NICE (the National Institute for Health and Care Excellence) produces a range of advice and guidance on medical practice and technologies. NICE was established in 1999, and in 2009 set up its Medical Technologies Evaluation Programme. This assesses new devices in terms of whether their use would offer benefits to the patient and NHS at a lower cost compared with current practice, or increased benefits for equal cost. NICE evaluates single products only, as multiple product assessments are time-consuming and mean that manufacturers have to wait longer for NICE to produce guidance on adoption of their technologies. Research into devices and diagnostics is often sparse and of low quality as there is little regulation requiring good research in this area. As a result, products are often not accepted for evaluation, because the evidence base supporting their claimed benefits is so poor.

## Key points

• Technologies which involve the use of new procedures need first to be evaluated for NICE Interventional Procedures Guidance which assess the safety and efficacy of procedures.

• In order for their product to receive a positive recommendation in NICE Medical Technologies Guidance manufacturers must show that their device provides advantages for patients and/or for the service, without increasing costs.

• Submissions need to be accompanied by enough evidence to support the claims.

• Selection of new devices submitted to the NICE Medical Technologies Evaluation Programme is based on the proffered evidence context for use and a plausible cost model.

• NICE also takes into account both expert advice and patient opinion when evaluating a new device.

• NICE Medical Technologies Guidance focuses on individual technologies but aims to influence and improve practice overall.

## NICE guidance

In the UK there are various programmes run by NICE which provide guidance on medical practices and technologies. These include:

• The Technology Appraisal Programme which produces guidance based on the clinical and cost effectiveness of pharmaceuticals and technologies.

• The Clinical Guidelines Programme which produces guidelines about the management of particular conditions.

• The Interventional Procedures Programme which looks at the safety and efficacy of new procedures in medicine and surgery, and produces guidance on their use.

• The Public Health Programme which produces guidance on preventing ill-health.

• The Medical Technologies Programme which looks at new devices and diagnostics, and produces guidance on their adoption.

• The Diagnostic Assessment Programme which evaluates complex and costly diagnostics.

## CardioQ

This technology is of special relevance to anaesthetists and has stimulated the interest of the anaesthetic community in the National Institute for Health and Care Excellence (NICE) Medical Technologies guidance. The guidance on the CardioQ oesophageal Doppler monitor [1] stated that the case for adopting CardioQ was supported by evidence of reduced complications, reduced use of central lines and shorter hospital stays. The guidance says that it should be considered for major high-risk surgery, where otherwise invasive or intravenous monitoring would be required.

## Origins of the Medical Technologies Programme

The Darzi review of the NHS published in 2008 emphasised a need to simplify the way new technologies were taken up across the NHS. As a result of this, NICE was charged with setting up a programme to encourage adoption of new devices and diagnostics [2]. The aims of the programme were to identify promising new technologies, evaluate them using the NICE methods [3], and then to promote their adoption, if appropriate.

In setting up the Medical Technologies Evaluation Programme, NICE involved a wide variety of interested parties including clinicians of all types, different patient groups, representatives of the medical device industry, commissioners (payers), health service managers and the Department of Health (government). A number of basic, broad principles were agreed for the development of a very meticulous process. These included:

• The programme should look at novel technologies, but not to the exclusion of technologies that have been around for some time but which have not become widely used;

• It should compare new technologies against current management;

• The evaluation process should involve two stages: first deciding whether a technology seems worth evaluating, and then evaluating it in detail.

For positive Medical Technologies guidance on adoption, the device should not be clinically inferior to current management, it should be at least as good as or better than the current practice, and it should be equal or less in cost compared with current management. The guidance should be based on single technologies and products, because multiple technology assessments take much longer. Finally, the programme should use a ‘cost consequences’ model in order to determine whether a new technology is likely to be cost saving and by how much.

## Submitting a new technology for NICE evaluation

Manufacturers can get any device or diagnostic straight onto the market with the normal regulatory requirement of a CE mark and scrutiny by the Medicines and Healthcare products Regulatory Agency (MHRA). However, if the device involves a new procedure, the safety and efficacy of that procedure must be reviewed by the NICE Interventional Procedures Committee. By contrast, involvement of manufacturers with the NICE Medical Technologies Evaluation Programme is entirely on a voluntary basis, but it potentially gives them a major advantage in terms of adoption of their technology, as seen with CardioQ.

Manufacturers need to produce evidence, particularly comparative and clinical utility evidence, not only showing that their product works, but that it works in everyday practice. They must provide as much clinical evidence as possible on the advantages to patients and/or the service, as well as defining current management practices which their technology would replace or alter. They must outline clear value propositions to allow cost modelling based on plausible assumptions. In their initial notification manufacturers are required to answer several questions in a limited number of words. They need to provide a clear bulleted list of claims of advantage compared with current management in patient outcomes or experience; system benefits; and any advantage for the sustainability (energy saving) agenda.

Factors taken into account in selecting a technology for detailed evaluation include:

• Improved patient outcomes, quality of life measures or survival rates;

• Less hospital visits and shorter hospital stays;

• Treating people as outpatients rather than inpatients;

• Better use of resources in terms of hospital facilities;

• Less staff time required when the technology is used;

• Other contributions to cost reduction, including capital costs and avoidance of treating complications;

• Less waste or less costs from transport or other energy use.

The value proposition needs to make clear whether use of the technology is being proposed in the context of primary or secondary care: either may be possible, but the value propositions may be radically different. Therefore manufacturers must consider the care pathway and make a clear description of the context for which their product is intended. Failing to provide a cost model with plausible assumptions is likely to result in a product not being selected for evaluation.

## The decision to evaluate a technology

The NICE process is based on two steps. The committee first sees the technologies on the basis of a briefing note, at which point it has relatively limited information on the product. Based on this and on the advice of experts, a decision is made whether to select the product for full evaluation. If a product is selected for evaluation, the Medical Technologies Advisory Committee then has a number of options. If the technology appears likely to have high impact associated with increased costs, it may be referred for a NICE Technology Appraisal. If the product involves a brand new procedure, it will be referred to NICE Interventional Procedures for evaluation. If it is a complex diagnostic – in other words for which there are several alternatives, such as one for which complex analysis of clinical outcomes will be required, or one which is likely to increase costs – then it is sent to the Diagnostics Committee. The most common scenario is one in which a device has claims which can be evaluated by the Medical Technologies Advisory Committee, which will then produce guidance on its adoption by the health service.

If a product is not selected for evaluation, the manufacturer is sent a letter which includes some of the reasons and which may offer advice about the evidence which would be needed to support evaluation of the product.

## The NICE evaluation process for medical technologies

If a technology is selected for evaluation, the manufacturer then provides the committee with a far more detailed submission. This submission and the manufacturer’s cost model are then analysed and critiqued independently by one of four external academic assessment centres contracted by NICE.

NICE’s independent Medical Technologies Assessment Committee, which produces the guidance, is heavily dependent upon expert advice. Some experts are nominated by the manufacturer and others by specialist societies. NICE also requests advice from patients and patient organisations, and occasionally a patient will come to the committee to give their personal evidence where relevant. There is also a wide range of experts on the committee who provide specialist knowledge in various different areas.

When guidance has been drafted it is subject to public consultation via the NICE website. All NICE guidance goes through a month of public consultation, receiving varying amounts of feedback. Every single comment from the public is considered by the Committee. Public consultation is a very important part of the evaluation process and sometimes results in extensive revisions of the guidance. After further checking by NICE, the guidance is published as NICE Medical Technologies Guidance.

## Limitations of the evidence

One problem with developing this kind of guidance is that the evidence on devices and diagnostics, compared to pharmaceuticals, is typically sparse and often poor in quality. This is because, unlike with drugs, there is little regulatory demand for good research on technologies. Another factor is that the UK MedTech industry comprises around 1,000 large companies and 3,000 smaller enterprises, many of which have limited resources and limited experience in research. Furthermore, because NICE is usually evaluating technologies at an early stage of their market life, the amount of research (especially related to any long term outcomes) may be very sparse. As a result of all these considerations NICE takes a permissive approach to the evidence, which may include use of data from audits, conference abstracts and unreported technical studies, in addition to peer reviewed publications.

## What has NICE achieved so far?

NICE has published a number of anaesthesia-related guidelines and CardioQ is a recent example. NICE guidance states that using the CardioQ device results in fewer complications, shorter hospital stays and quicker recovery. Using a complex cost model, which takes into account the costs of any changes in the care pathway where necessary, NICE has estimated a saving of around £1,100 per patient by changing to CardioQ [1].

Another technology recommended for adoption in NICE Medical Technologies guidance is the Inditherm warming mattress [4], which helps to prevent perioperative hypothermia. There was sufficient evidence to support the claim that the Inditherm mattress may produce fewer complications and is easier to use than forced air warming. NICE calculated that adopting this technique instead of forced air warming could save approximately £11,000 per operating theatre [5].

NICE guidance focuses on single products, but it also aims to influence practice overall. For example, it is hoped that the NICE guidance on CardioQ will help to influence the overall management of perioperative fluid balance by monitoring cardiac output.

## Potential risks to manufacturers

Not all of NICE guidance is positive. For example, a device designed to give photodynamic therapy at home for small skin tumours had very limited evidence, and its adoption was therefore not recommended in the guidance [6]. Manufacturers may reasonably fear that if guidance does not support their device because of poor evidence it will have a negative effect on sales of their product and perhaps on their reputation. They can avoid this risk by providing a reasonable amount of good supporting evidence for their product. Indeed, one aim this kind of NICE’s medical technologies guidance is to encourage better evidence on devices and diagnostics. It is important to note that negative guidance does not mean that a product cannot be used and it may be suitable for a future evaluation if the evidence base is improved.

Sometimes technologies which seem promising are notified to NICE, with some supporting evidence but their capacity to work in practice in an NHS context and to deliver the claimed benefits, is uncertain. Alongside the Medical Technologies Programme, NICE has created a means of facilitating research in a UK setting. This involves creating partnerships between manufacturers and appropriate academics and health service researchers. After further research has been undertaken, the product is re-evaluated for NICE guidance.

NICE’s contracted external academic centres engage with manufacturers to facilitate this research. These external assessment centres translate the submission into a research protocol, introducing manufacturers to interested academic centres and hospitals in the UK. Once the research is completed, the external assessment centre then analyses it in order to see whether the relevant questions have been answered. This analysis is independent of the manufacturers, even though they may have funded the research, in part or in full. An example is a new trial resulting from NICE recommendations, which is testing a new form of ultrasound treatment delivered through a saline mist with the aim of enhancing the healing of chronic wounds [5].

It is hoped that in the long-term, the pressure to proffer adequate evidence may help to influence manufacturers to conduct more and better clinical trials of new devices. NICE is currently in the process of setting up a system similar to its scientific advice service for drugs, whereby companies pay a modest sum in return for advice about how to produce appropriate evidence.

## Conclusions and future NICE developments

NICE Medical Technologies guidance influences practice by making explicit recommendations based on the available evidence and by providing clear, balanced judgements. Importantly, the guidance outlines the expected advantages of a technology and the context in which they can be expected. It clearly describes the cost consequences of using the technology instead of current management, taking into account all aspects of cost, including possible changes to care pathways. NICE’s guidance provides a comparison between the advantages and cost benefits of new technologies compared with current methods of practice, in order to reassure commissioners and managers about the overall benefit that new technologies offer to patients and to the NHS.

## Abbreviations

NICE: National Institute for Health and Clinical Excellence; MHRA: Medicines and Healthcare products Regulatory Agency.

## Competing interests

The author declares that he has no competing interests.
